# Parental provisioning behaviour plays a key role in linking personality with reproductive success

**DOI:** 10.1098/rspb.2013.1019

**Published:** 2013-08-07

**Authors:** A. Mutzel, N. J. Dingemanse, Y. G. Araya-Ajoy, B. Kempenaers

**Affiliations:** 1 Department of Behavioural Ecology and Evolutionary Genetics, Max Planck Institute for Ornithology, 82319 Seewiesen, Germany; 2 Evolutionary Ecology of Variation Research Group, Max Planck Institute for Ornithology, 82319 Seewiesen, Germany; 3 Department of Biology II, Behavioural Ecology, Ludwig Maximilians University of Munich, 82152 Planegg-Martinsried, Germany

**Keywords:** aggression, behavioural syndrome, exploration, parental care, path model, structural equation modelling

## Abstract

Repeatable behavioural traits (‘personality’) have been shown to covary with fitness, but it remains poorly understood how such behaviour–fitness relationships come about. We applied a multivariate approach to reveal the mechanistic pathways by which variation in exploratory and aggressive behaviour is translated into variation in reproductive success in a natural population of blue tits, *Cyanistes caeruleus*. Using path analysis, we demonstrate a key role for provisioning behaviour in mediating the link between personality and reproductive success (number of fledged offspring). Aggressive males fed their nestlings at lower rates than less aggressive individuals. At the same time, their low parental investment was associated with increased female effort, thereby positively affecting fledgling production. Whereas male exploratory behaviour was unrelated to provisioning behaviour and reproductive success, fast-exploring females fed their offspring at higher rates and initiated breeding earlier, thus increasing reproductive success. Our findings provide strong support for specific mechanistic pathways linking components of behavioural syndromes to reproductive success. Importantly, relationships between behavioural phenotypes and reproductive success were obscured when considering simple bivariate relationships, underlining the importance of adopting multivariate views and statistical tools as path analysis to the study of behavioural evolution.

## 1. Introduction

Meta-analyses have revealed that behavioural traits typically show substantial individual repeatability [1], and that individuals from the same population also vary in suites of correlated behaviours [2]. For example, individuals that are relatively active also tend to be relatively explorative and aggressive compared with less-active individuals from the same population [3]. The occurrence of such between-individual variation in single behaviours over time or across contexts is now commonly called ‘animal personality’ in the behavioural ecology literature [4,5], while the term ‘behavioural syndrome’ [6,7] refers to non-zero behavioural correlations between individuals [8]. Theoreticians have provided various explanations for why such patterns of between-individual behavioural (co)variance might result from natural selection [9–11], though few studies have yet explicitly tested their predictions and assumptions (but see [12,13]). As a consequence, we still know relatively little about why personalities and syndromes persist in nature.

In natural populations, repeatable behavioural traits similar to exploratory tendency or aggressiveness covary with proxies for fitness such as survival [14–16] or reproductive success [12,16–19], implying that behavioural phenotypes are subject to natural selection. Yet, our insight in how behavioural phenotypes affect fitness is still limited because most studies to date have estimated fitness effects of single components of syndromes, e.g. only exploratory behaviour [14,18] or only aggressiveness [20], rather than asking which components of behavioural syndromes are directly versus indirectly under selection via their correlations with other behaviours. Moreover, we know relatively little about mechanistic pathways by which repeatable behaviour is translated into fitness, since those pathways are typically being implied rather than measured explicitly [21]. For example, is exploratory behaviour associated with reproductive success because it affects the acquisition of high-quality territories [17,22], or rather because it is associated with responsiveness towards variation in food resources [23,24] or offspring demands [25]? And which of these associations are directly due to exploratory tendency rather than representing indirect effects of aggressiveness? Such questions illustrate the necessity for integrative studies where distinct direct and indirect pathways by which components of correlated behaviours might affect fitness are simultaneously quantified [6]. This study aimed to investigate how two key behavioural traits that are often structured in a behavioural syndrome, namely aggressiveness and exploratory behaviour, affect reproductive success and how such fitness associations might come about. We therefore considered, based on previous literature, various direct and indirect pathways (detailed below), and used data collected for a passerine bird with bi-parental care, the blue tit, *Cyanistes caeruleus*, to assess the relative level of support for each.

**Figure 1.**
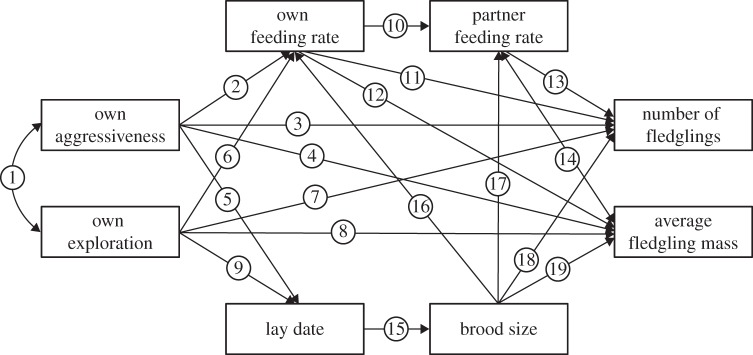
Hypothesized path model for males. One-headed arrows indicate the direction of hypothesized causal links. Double-headed curved arrows indicate simple hypothesized correlations. Path numbers are given in circles. We constructed the same path model for females with the exception that aggressiveness was not included because this trait was not assayed for this sex.

We simultaneously considered the following pathways by which components of the aggressiveness–exploration syndrome would translate into reproductive success (figure 1). First, we expected that aggressiveness negatively affects feeding rate in males (path 2) [26,27], because aggressive males are generally thought to trade-off investment in offspring provisioning with investment into nest and territory defence [28,29]. Provisioning effort should also be affected by brood size (paths 16 and 17) [30], and individual parents are expected to compensate for changes in partner provisioning effort (path 10) [31]. Provisioning rates in turn should positively and directly translate into reproductive success (paths 11–14) [32]. Taken together, these relationships were expected to result in an indirect link between male aggressiveness and number (and condition) of fledglings via male and female provisioning effort.

Second, fast exploratory behaviour has been implied to positively affect the ability to acquire high-quality territories in males [17,33], and females in such territories typically lay earlier in the season than those breeding on low-quality territories [34]. We therefore expected a negative effect of male exploratory behaviour on the lay date of his social mate (path 9), whereas lay date should negatively influence brood size (path 15) [35]. Brood size, in turn, typically negatively affects fledgling weight because feeding frequency per nestling will decline with increasing brood size (path 19) [36] and was expected to positively affect the number of fledglings (path 18) [35,36]. Provided that relationships between male exploratory behaviour and acquired territory quality reported in the literature [17,22] were in fact indirect and causally mediated by aggressiveness, these relationships are expected to create a second indirect causal pathway between male aggressiveness and reproductive success via lay date (path 5) and brood size. In other words, we also expected that exploratory behaviour and aggressiveness were structured in a behavioural syndrome (path 1) [3].

For females, we do not have a formal literature-based framework of hypotheses concerning the link between exploratory behaviour and lay date. However, fast-exploring females might also be relatively aggressive and win from slow-exploring females in spring, when competing for territorial males. In line with this, fast-exploring great tit, *Parus major*, females tended to be more dominant at clumped winter food resources than slow-exploring ones [33]. Similarly, it has been shown previously that female–female aggression plays an important role in competition for breeding opportunities in blue tits [37]. We therefore hypothesized that fast-exploring females are able to acquire high-quality territories, and initiate their clutches earlier compared with slow-exploring females (resulting in a negative causal link between female exploratory tendency and lay date; path 9).

Third, we investigated how exploratory behaviour related to provisioning rates (path 6). This relationship has rarely been studied [25,38], but exploration has been directly linked to foraging abilities [24,39,40], and reproductive success in passerine birds, both in the wild [14,17–19] and in the laboratory [41]. Therefore, we predicted, for both males and females, a pathway linking exploratory behaviour with reproductive success via their own and their mate's provisioning rate (paths 6 and 10–14), with slow-exploring parents providing more parental care and therefore having a higher reproductive success than fast explorers [17].

Finally, we considered that aggressiveness and exploratory behaviour might also directly or indirectly affect reproductive success via unknown (i.e. not yet hypothesized) behavioural pathways. Thus, we tested direct (i.e. residual) pathways linking aggressiveness (or exploratory behaviour) with the proxies for fitness (paths 3, 4, 7 and 8).

## 2. Material and methods

### (a) Study site and general field procedures

The study was carried out in a nest-box population of blue tits, in southern Germany (Westerholz, 48°08′ N, 10°53′ E) (detailed in [42]), during the breeding seasons of 2009 and 2011. From early March till the end of the breeding season, nest-boxes were checked at least once per week. We recorded lay date (date of the first egg) and hatching and fledging date, as well as the number of hatchlings (brood size) and fledglings. When nestlings were between 9 and 10 days old, both parents were caught inside the nest-box. Unbanded birds obtained a numbered metal band and a unique combination of three colour bands and were equipped with a uniquely coded passive integrated transponder (PIT) tag (EM4102 ISO animal tag 134.2 kHz ISO, 8.5 × 2.12 mm, 0.067 g), following Nicolaus *et al.* [43]. PIT tags enabled automatic monitoring of provisioning behaviour of tagged parents without direct observation of nest-boxes. At day 14, when nestlings reached their final fledging weight and size, weight and tarsus length of nestlings were measured. The number of fledglings and average fledgling weight was used as a measure of reproductive success. As parents have to trade-off investment in number versus quality (i.e. weight, [36]) of fledglings, these two measures combined represent a good proxy for reproductive success.

### (b) Aggression tests

In both years of study, male aggressiveness was measured once between March and May by subjecting pairs to a simulated territorial intrusion. Final sample sizes for 2009 and 2011 were 31 and 58, respectively. A taxidermic mount of a male blue tit on a 1.5 m long wooden pole was placed 2 m from the focal nest-box. A small loudspeaker (Radio shack, Mini Audio Amplifier) connected to a MP3 player for song playback was fixed directly underneath. A snap trap was fixed on top of the pole to catch attacking males landing on the trap. Aggression tests were conducted at nest-boxes where birds had been registered frequently (either a PIT-tagged male used the box for sleeping during winter or a pair occupied the box at the onset of the breeding season). After starting song playback, the behaviour of the male territory holder was observed from a distance of 15 m until the bird was caught or for a maximum of 30 min. In total, we used six male blue tit models and five blue tit songs, randomly assigned to aggression tests to prevent pseudo-replication [44]. Neither song nor model identity affected male aggressiveness (see the electronic supplementary material, text S1). Only observations of individuals that were identified (caught during the aggression test or identified by reading colour band combinations) as the male later feeding at the focal nest (89 out of 121 tested males) were included for later analyses.

As proxy for male aggressiveness we used approach latency (for rationale, see electronic supplementary material, text S2), multiplied by −1 to obtain a continuous variable where higher values represented increased aggressiveness. We performed a box-cox transformation, resulting in models with residuals not deviating from a Gaussian distribution. Because nest stage during the aggression test significantly affected male aggressiveness (see the electronic supplementary material, table S1), this variable was controlled for in all subsequent analyses by expressing each aggression score as the deviation from the mean value for each nest stage.

### (c) Exploration tests

Exploratory behaviour was measured in spring 2009 and 2011, using a cage test adapted from the classic ‘novel environment test’ [24,45]. Birds were captured with a snap trap during the aggression test and/or inside the nest-box when feeding nestlings (see above) and immediately brought to a car fitted with the exploration cage in the back (see the electronic supplementary material, figure S1). The behaviour of the subject was recorded for 5 min with a video camera (JVC Everio GZ-MG77E) placed 1.5 m from the cage. An individual's movements between different cage locations were later scored from videos with an event recorder (The Observer v. 5.0.31, Noldus Information Technology, The Netherlands). The total number of hops and flights within and between different locations (see the electronic supplementary material, figure S1) was used as a proxy for exploratory behaviour following the procedure outlined elsewhere [45] for the classic novel environment test in wild great tits. Repeated measures of the same individuals were used to calculate repeatability. For all other analyses, we used measures obtained during adult catching. Because nearly all parents were caught with this method, this ensured that our dataset represented an unbiased subsample of the study population.

### (d) Parental provisioning behaviour

In 2011 alone, provisioning behaviour was observed at 48 nest-boxes with automatic nest-box recording devices (see the electronic supplementary material, text S3) as part of another experiment detailed elsewhere [46]. Here, we only used ‘control’ data of that experiment, recorded the day before and the day after the experimental treatment. For each nest, we extracted 90 min of feeding data per day on 2 days when nestlings were 11 and 14 days old. An individual's average feeding rate per hour across both observation days was used as a measure of provisioning behaviour.

### (e) Statistical analyses

#### (i) Repeatability

Although the repeatability of exploratory behaviour and feeding rate has been demonstrated before [45,47], it has not yet been quantified for our population nor for our specific exploration test. Repeatability of both behavioural traits was calculated using univariate mixed-effect models fitted in the MCMCglmm package [48] of R v. 2.14.2 [49]. For exploratory behaviour random intercepts were fitted for individual identity, while sex and test sequence were included as fixed effects. We ran models for both years separately and for both years combined with year as additional fixed effect. To calculate repeatability of feeding rate across the two observation days, we fitted random intercepts for individual identity and sex as a fixed effect. Repeatability was calculated as the between-individual variance divided by the sum of the between-individual plus residual (i.e. within-individual) variance not accounted for by the fixed effects [50].

#### (ii) Path analyses

Path analysis [51] was applied to infer how proxies for short-term fitness were directly versus indirectly related to behavioural traits (i.e. aggression and exploration), parental investment (provisioning rate) and female reproductive decisions (lay date and clutch size). Since provisioning data were not available for 2009, only data from 2011 were included. We estimated the variance–covariance matrix between all hypothesized predictor and response variables and took the estimated matrices forward for path analyses. We ensured that the uncertainty around the estimates was appropriately taken forward by applying a Bayesian framework with Markov chain Monte Carlo (MCMC) methods. Variances and covariances were derived by fitting two multivariate models (MCMCglmm package; see electronic supplementary material, text S4 for details on prior specifications), one for each sex, with aggression (males only), exploratory behaviour, lay date, brood size, the focal individual's and partner's feeding rate, and fledging number and weight as response variables (see the electronic supplementary material, tables S2 and S3). Excluding aggressiveness from the male model, to make it directly comparable to the female model, did not result in any changes in the model outcome (results not shown).

The key advantage of the MCMC method is that the model output gives the entire posterior distribution of each fixed and random parameter. This distribution can subsequently be used for further analyses, such that the uncertainty around point estimates is appropriately taken forward. Path analyses were performed within the structural equation modelling package in R [52], where we applied a Bayesian framework by running each of the specified path models once for each of the 1000 estimated variance–covariance matrices (see above). Using these 1000 runs, we calculated for every specified path the most likely path coefficient value and its associated 95% credible interval. Credible intervals not including zero indicate statistical significance. For intervals only slightly overlapping zero, we calculated how often the estimate was positive or negative, thus giving a value that is comparable with a *p*-value [53]. Path coefficients for compound paths were estimated to infer the level of statistical support for indirect effects which was achieved by multiplying all coefficients along the focal path [51]. Path analysis allows for the calculation of partial correlation coefficients between two variables while simultaneously controlling for effects of all other variables in the model [51]. This makes it a powerful tool to disentangle direct from indirect effects (i.e. produced by the effect on another correlated variable). This is of particular importance for datasets where predictor variables are assumed to be highly inter-correlated [51], as in behavioural syndrome studies (see also [54]). As paths between the variables were hypothesized *a priori*, we present the results for the full model, which also includes the paths not supported by the model [55,56].

## 3. Results

### (a) Repeatability of behaviour

Exploratory behaviour was repeatable in 2011 (*R* = 0.60, 95% CI: 0.13, 0.79), and also when data of both years were combined (*R* = 0.66, 95% CI: 0.44, 0.76; the statistical model did not converge for 2009; electronic supplementary material, table S4). Feeding rate was significantly repeatable across the two observation days (*R* = 0.78, 95% CI: 0.64, 0.83; electronic supplementary material, table S4). These findings imply that individuals consistently differed in how they explored a novel environment and how often they fed their offspring.

### (b) Parental feeding rates, breeding decisions and reproductive success

**Figure 2.**
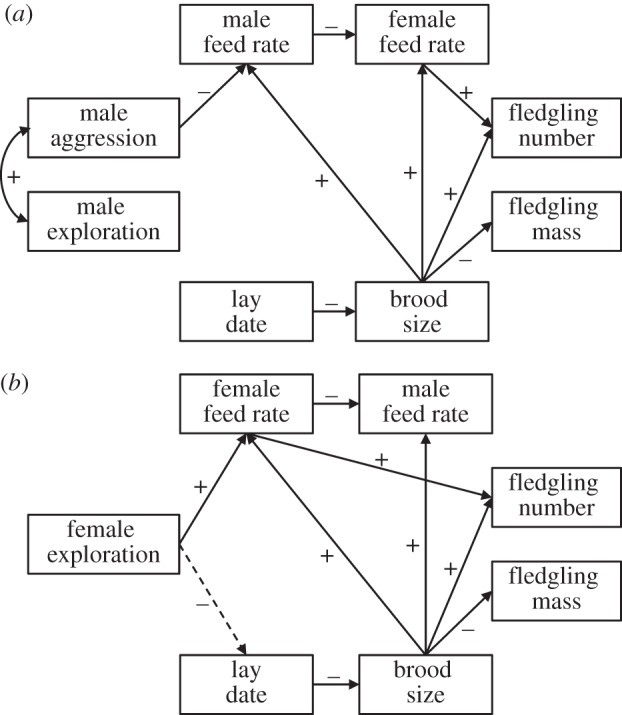
Supported paths in models for (*a*) male and (*b*) female blue tits. Only paths with considerable statistical support are shown. Black arrows indicate strong support (credible intervals not overlapping zero), dashed black lines indicate some support (credible intervals slightly overlapping zero but with *p* < 0.05).

The variance–covariance matrices calculated separately for males and females were largely based on the same parental feeding rates, lay date, brood size and reproductive success data. Therefore, path models for both sexes revealed similar patterns for all pathways including these variables. There was strong support for a negative pathway linking an individual's and its partner's feeding rate (figure 2*a,b*; table 1, path 10), suggesting that low investment in offspring provisioning of one pair member was associated with a higher partner effort. Even though male feeding rate did not directly affect fledgling production (table 1, path 11 for male model, path 13 for female model), it indirectly and negatively affected fledging success via female parental effort (figure 2*a*,*b* and table 1, compound path A), implying that female provisioning behaviour was more important for nestling survival. The path model further provided strong support for a trade-off between offspring quality and quantity, mediated by brood size, which positively affected fledgling production both directly (figure 2*a*,*b* and table 1, path 18), and indirectly (via female feeding rate; figure 2*a*,*b* and table 1, compound path B). At the same time, brood size directly and negatively influenced average fledgling mass (figure 2*a*,*b* and table 1, path 19). Furthermore, the path model supported a significant negative pathway linking lay date with brood size (figure 2*a*,*b* and table 1, path 15), providing strong support for our hypothesis for a direct link between these variables: females initiating egg laying earlier also had larger broods.

**Table 1.** Estimated partial regression coefficients for male and female path models. The estimate of a path coefficient of a compound path (containing more than one path) is the product of the coefficients along its path. Bold numbers indicate path coefficients (path coef.) that are strongly supported by the model (95% credible interval CI not overlapping zero). Italic numbers indicate path coefficients that have some support from the model (credible intervals slightly overlapping zero but with *p* < 0.05).

path number	hypothesized link	males			females		
		*n*	path coef.	95% CI	*n*	path coef.	95% CI
1	aggression → exploration	41	**0.27**	0.01, 0.54	—	—	—
2	aggression → own feed rate	33	**−0.49**	−0.64, −0.09	—	—	—
3	aggression → fledgling no.	45	−0.06	−0.25, 0.17	—	—	—
4	aggression → fledgling mass	44	−0.27	−0.52, 0.03	—	—	—
5	aggression → lay date	43	0.23	−0.12, 0.52	—	—	—
6	exploration → own feed rate	44	0.22	−0.17, 0.38	37	**0.55**	0.12, 0.68
7	exploration → fledgling no.	60	0.10	−0.11, 0.23	56	−0.12	−0.29, 0.08
8	exploration → fledgling mass	59	0.03	−0.21, 0.24	55	−0.07	−0.32, 0.22
9	exploration → lay date	58	−0.20	−0.54, 0.12	54	*−0.38*	−0.62, 0.02
10	own feed rate → partner feed rate	48	**−0.59**	−0.83, −0.32	48	**−0.56**	−0.76, −0.35
11	own feed rate → fledgling no.	48	0.19	−0.14, 0.45	48	**0.52**	0.24, 0.74
12	own feed rate → fledgling mass	48	−0.14	−0.47, 0.32	48	0.23	−0.17, 0.59
13	partner feed rate → fledgling no.	48	**0.43**	0.22, 0.69	48	0.17	−0.10, 0.44
14	partner feed rate → fledgling mass	48	0.16	−0.20, 0.46	48	0.03	−0.42, 0.35
15	lay date → brood size	67	**−0.52**	−0.64, −0.25	71	**−0.50**	−0.69, −0.34
16	brood size → own feed rate	48	**0.41**	0.17, 0.61	48	**0.66**	0.46, 0.84
17	brood size → partner feed rate	48	**0.56**	0.38, 0.80	48	**0.29**	0.03, 0.50
18	brood size → fledgling no.	69	**0.52**	0.29, 0.72	73	**0.53**	0.32, 0.75
19	brood size → fledgling mass	66	**−0.53**	−0.85, −0.27	69	**−0.47**	−0.82, −0.18
compound path	individual path numbers						
A	10 × 13		**−0.28**	−0.46, −0.09		—	—
B	17 × 13 (males); 16 × 11 (females)		**0.28**	0.14, 0.44		**0.10**	0.02, 0.26
C	2 × 10 × 13		**0.09**	0.01, 0.23		—	—
D	1 × 2		*−0.06*	−0.26, 0.02		—	—
E	6 × 11		—	—		**0.15**	0.02, 0.39
F	9 × 15		—	—		**0.10**	0.02, 0.26
G	9 × 15 × 18		—	—		*0.09*	−0.01, 0.19
H	9 × 15 × 19		—	—		*−0.06*	−0.21, 0.02

### (c) Male behaviour and reproductive success

The path model for males supported a link between aggressiveness and exploratory behaviour, with more aggressive individuals exploring a novel environment faster than less aggressive ones (figure 2*a* and table 1, path 1). At the same time, there was a negative link between aggressiveness and male feeding rate, with aggressive males feeding their offspring at a relatively low rate (figure 2*a*; table 1, path 2). The model further revealed an indirect positive effect of male aggressiveness on fledgling production via male and female feeding rates (table 1, compound path C).

The link between male aggressiveness and female lay date was not supported by the path model (table 1, path 5), thereby failing to confirm the hypothesis that aggressive males derive fitness benefits from having higher quality territories (or mates). There was no evidence for any unknown pathway, since the direct (i.e. ‘residual’) pathways between aggressiveness and reproductive success (table 1, paths 3 and 4) were not (strongly) supported.

To assess whether there was an overall effect of male aggressiveness on reproductive success, we investigated the raw phenotypic correlations derived from the multivariate model specified earlier. We found no support for an overall effect of male aggressiveness on fledgling production (*r* = 0.06, 95% CI: −0.28, 0.27; electronic supplementary material, table S2). At first glance, this result might seem surprising, as the correlation should reflect the sum of all path coefficients and the path model only supported a positive indirect link between aggression and fledgling production mediated via parental feeding rates. There are two explanations. First, the correlation between aggressiveness and fledgling production was not zero, but was not detected due to lack of power (type II error). Yet, the estimate was close to zero and its credible interval was largely overlapping zero, making this explanation unlikely. Second, other counteracting pathways might have been present but were not strongly supported because their effects were subtle, inherently resulting in a lack of power given the data at hand. This explanation is more likely, since all other paths indeed had negative point estimates, suggesting that aggression might have negatively affected fledgling production via female lay date, male feeding rate and an unknown (residual) pathway, jointly cancelling out the positive effect of indirect pathways via the birds' provisioning rates.

Finally, between male variation in exploratory behaviour did not directly affect male provisioning rate (table 1, path 6) or female lay date (table 1, path 9). The negative indirect link between exploratory and provisioning behaviour, mediated by the covariance between exploratory behaviour and aggression was weak, but significant (*p* = 0.04; table 1, compound path D). There was little support for an unknown pathway, since direct (i.e. ‘residual’) pathways between exploratory behaviour and reproductive success were not supported (table 1, paths 7 and 8). In other words, in this population, links between exploratory behaviour and reproductive parameters are likely solely caused by its correlation with aggressiveness, implying that exploratory behaviour was an indirect rather than a direct target of selection.

### (d) Female behaviour and reproductive success

In females, there was support for two distinct indirect effects of exploratory behaviour on reproductive success. First, fast-exploring females fed their nestlings at a relatively high rate (figure 2*b* and table 1, path 6), resulting in a positive indirect effect of female exploratory tendency on fledgling production (table 1, compound path E). Second, female exploratory behaviour affected the timing of reproduction, with fast explorers initiating clutches relatively early in season having larger broods (table 1, compound path F), resulting in a slight positive effect on fledgling production and a slight negative effect on fledgling mass (*p* = 0.04, table 1, compound paths G and H). Even though there was no strong support for an overall effect of female exploratory behaviour on fledgling production, the positive correlation coefficient between these variables (*r* = 0.18, 95% CI: −0.13, 0.35; electronic supplementary material, table S3) suggests that such a link remained undetected due to a lack of power (see above).

## 4. Discussion

We showed that aggressive males fed their nestlings at a lower rate than less aggressive ones. Their low feeding rate was associated with an increased female effort resulting in a positive effect on fledgling production. Our study also revealed a sex-specific link between exploratory behaviour and reproductive success. Fast-exploring females, but not males, had a higher fledging success than slow-exploring ones, mediated by both a higher feeding effort and an earlier lay date.

### (a) Aggressiveness and reproductive success

As expected, aggressive males fed their offspring less often than non-aggressive ones. There was nevertheless a positive effect of male aggressiveness on fledgling production mediated by parental feeding rates. This was because low male provisioning effort was directly associated with an increased female effort, which in turn positively affected fledgling production. There are several explanations for this finding. First, the behavioural trade-off between different aspects of parental care might be resolved by a division of labour between members of a pair. For instance, aggressive males might invest more time and energy in territory or nest defence [26,57], thereby increasing offspring survival [58], whereas their female partners mainly focus on offspring provisioning. To test this hypothesis, it would be necessary to not only measure feeding rate but also other aspects of parental care, such as nest defence behaviour. Second, as parental care is costly [32], both members of a pair benefit from investing less than the partner, creating a conflict between parents over care [31,59]. This conflict is assumed to lead to a negotiation between partners where an individual benefits from adjusting its care directly in response to its partner [60]. Aggressive males might be better in ‘winning’ this conflict, reducing their own investment in parental care at the expense of their female partner. However, the outcome of this conflict is predicted to be evolutionarily stable only if the partner partially compensates for the shortfall of the other pair member [31,60,61]. In our study, females instead overcompensated for low feeding rates of aggressive partners, implying that this hypothesis on its own cannot explain these findings. Third, aggressive males could provide females with other benefits apart from help with offspring provisioning. For instance, aggressive males might be of higher genetic quality, passing on the good genes to their offspring. As such high-quality offspring are more valuable, females paired with these males might be willing to increase investment in current at the expense of future reproduction [62,63]. Aggressive males might also be better in acquiring and defending high-quality breeding territories [17], facilitating foraging for females and thus permitting males to reduce their own investment in offspring provisioning. However, male aggression was not associated with female lay date, suggesting that male territory did not play an important role. Overall, our study indicates that division of labour as well as male quality might be involved in mediating the link between aggression and reproductive success. Yet, experiments are now needed to test the predictions of these hypotheses.

### (b) Exploratory behaviour and reproductive success

Male exploratory behaviour and feeding rate were not associated, thereby contradicting repeated suggestions that exploratory behaviour affects parental care [17,25]. Possibly, male exploratory behaviour, rather than impacting feeding rates, affects other aspects of provisioning behaviour that were not included in our study, such as prey type or load size [64]. For example, there is individual variation in both visit rate and load size in blue tits [65]. The finding that male—in contrast to female—feeding rate did not affect fledgling production must imply that male feeding rate alone was not a good predictor of the amount of food brought to the nest. For instance, fast-exploring males might cover larger distances while foraging [23], thus reaching less depleted food patches with more profitable (e.g. larger) prey items [66]. Alternatively, they might be more selective in prey choice, thus spending more time searching for food. Both options would lead to fast explorers visiting the nest less often, but delivering larger prey items. However, if larger prey sizes would completely compensate for lower feeding rates, one would not expect the demonstrated negative link between male and female feeding rates. It thus remains to be tested whether variation in prey size plays an important role in determining provisioning efforts in male blue tits.

Female exploratory behaviour was a good predictor of feeding rate. Fast-exploring females fed their offspring at a higher, not at the expected lower rate, than slow-exploring ones. At the same time, female feeding rate positively affected fledgling production, indicating that, at least in females, feeding rate was a good predictor for the total amount of food brought to the nest and that other aspects of provisioning behaviour played a less important role compared with males. Earlier work showed that slow explorers were more flexible and better at locating new food sources, and therefore adapted more easily to changing and harsh environmental conditions [24,39]. Our study population breeds in a high-quality habitat (mature oak forest) and nesting success in the year of the study was relatively high, indicating that the caterpillar peak matched well with nestling feeding peak. Under such favourable environmental conditions, fast explorers might forage more efficiently than slow-exploring birds [14,67], allowing them to provision their offspring at a higher rate. The finding that fast-exploring females invested more in parental care also provides some support for current theoretical life-history trade-off models predicting that aggressive, fast-exploring and risk-taking individuals have moderate future fitness expectations and therefore invest more in current reproduction, whereas slow-exploring and relatively risk-averse individuals should have better future expectations and therefore invest less in current reproduction [68,69]. Possibly, this effect was not found in males, because other aspects of parental care, besides feeding rates, better indicate male investment in current reproduction. For instance, aggressive males might defend their territory or nest more vigorously than non-aggressive ones, thereby permitting females to concentrate on offspring provisioning.

### (c) Assortative mating and behavioural syndromes

Aggressive males, which were also fast explorers, had mates that fed their offspring at a high rate. At the same time, fast-exploring females generally fed their offspring more often. This could result in sexual selection favouring assortative mating with respect to exploratory behaviour, as has previously been suggested for great tits and other species [70]. In addition, it has been shown previously that assortatively mated pairs produced fledglings in the best condition [17,41] had a higher fledging success [71] and recruited more offspring than non-assortative pairs [14]. However, fitting male aggressiveness and male and female exploratory behaviour into a single path model did not reveal any covariance between male and female behaviour (results not shown), implying that the detected effects were not caused by assortative mating, but were rather caused by an individual's own behavioural phenotype.

Our study further revealed the expected positive relationship between male aggressiveness and exploratory behaviour. Such an aggression–exploration syndrome has previously been shown for a variety of taxa [7] and has been suggested to emerge when individuals of the same population use different behavioural strategies to cope with stressful situations [72]. This particular behavioural syndrome has been predicted to include behaviours related to parental care [25]. Interestingly, we showed that only aggressiveness, but not exploratory behaviour, was directly linked to nestling provisioning. This finding implies that despite their covariance, the two behavioural traits were nevertheless sufficiently distinct to allow detection of distinct behaviour-specific effects on reproductive success. Our results thus suggest the existence of at least two independent proximate mechanisms involved in driving the variation in these two behavioural traits. An alternative explanation would be that our measurement error is so substantial that any correlation between the two behaviours would be underestimated.

The present study focused on phenotypic correlations between individuals documented within a single season, implying that we cannot currently ascertain that the reported paths represent long-term between- as opposed to within-individual relationships [73]. Between-individual correlations result from differences in average behaviours between individuals caused by variation in genetic constitution and permanent environment effects, whereas within-individual correlations result from correlated plastic behavioural responses to environmental conditions [73]. As part of another study in great tits, we are therefore currently collecting data on the same individuals for multiple years, which allows us to partition pathways similar to those reported in the current study into between- and within-individual components [73]. Another consequence of our focus on a single year is that we were not able to evaluate whether the reported relationships (selection pressures) were general versus year-specific. Indeed, year-to-year variation in selection pressures acting on behavioural traits, such as aggressiveness, sociability and exploratory behaviour have been documented in a range of taxa [21]. Addressing the stability of the variance–covariance matrix and its associated underlying pathways will thus represent an exciting avenue for future research, and might shed light on the outstanding question of how animal personality variation is maintained in natural populations.

## 5. Conclusions

This paper revealed the mechanistic pathways by which aggressiveness and exploratory behaviour were affecting reproductive success. The reported mechanisms would have remained undetected if we had failed to apply a multivariate perspective on behavioural evolution [54,74], thereby illustrating the added value of holistic approaches towards the study of adaptive evolution [6].

## References

[1] Bell AM Hankison SJ Laskowski KL 2009 The repeatability of behaviour: a meta-analysis. Anim. Behav. 77, 771–783 10.1016/j.anbehav.2008.12.022 (doi:10.1016/j.anbehav.2008.12.022) PMC3972767 24707058

[2] Garamszegi LZ Markó G Herczeg G 2012 A meta-analysis of correlated behaviours with implications for behavioural syndromes: mean effect size, publication bias, phylogenetic effects and the role of mediator variables. Evol. Ecol. 26, 1213–1235 10.1007/s10682-012-9589-8 (doi:10.1007/s10682-012-9589-8)

[3] Verbeek MEM Boon A Drent PJ 1996 Exploration, aggressive behavior and dominance in pair-wise confrontations of juvenile male great tits. Behaviour 133, 945–963 10.1163/156853996X00314 (doi:10.1163/156853996X00314)

[4] Réale D Dingemanse NJ Kazem AJN Wright J 2010 Evolutionary and ecological approaches to the study of personality. Phil. Trans. R. Soc. B 365, 3937–3946 10.1098/rstb.2010.0222 (doi:10.1098/rstb.2010.0222) 21078646 PMC2992753

[5] Dingemanse NJ Kazem AJN Réale D Wright J 2010 Behavioural reaction norms: animal personality meets individual plasticity. Trends Ecol. Evol. 25, 81–89 10.1016/j.tree.2009.07.013 (doi:10.1016/j.tree.2009.07.013) 19748700

[6] Sih A Bell AM Johnson JC Ziemba RE 2004 Behavioral syndromes: an integrative overview. Q. Rev. Biol. 79, 241–277 10.1086/422893 (doi:10.1086/422893) 15529965

[7] Sih A Bell AM 2008 Insights for behavioral ecology from behavioral syndromes. Adv. Study Behav. 38, 227–281 10.1016/S0065-3454(08)00005-3 (doi:10.1016/S0065-3454(08)00005-3) PMC4075144 24991063

[8] Dingemanse NJ Dochtermann N Nakagawa S 2012 Defining behavioural syndromes and the role of ‘syndrome deviation’ in understanding their evolution. Behav. Ecol. Sociobiol. 66, 1543–1548 10.1007/S00265-012-1416-2 (doi:10.1007/S00265-012-1416-2)

[9] Dingemanse NJ Wolf M 2010 Recent models for adaptive personality differences: a review. Phil. Trans. R. Soc. B 365, 3947–3958 10.1098/rstb.2010.0221 (doi:10.1098/rstb.2010.0221) 21078647 PMC2992752

[10] Wolf M Weissing FJ 2010 An explanatory framework for adaptive personality differences. Phil. Trans. R. Soc. B 365, 3959–3968 10.1098/rstb.2010.0215 (doi:10.1098/rstb.2010.0215) 21078648 PMC2992748

[11] Dall SRX Houston AI McNamara JM 2004 The behavioural ecology of personality: consistent individual differences from an adaptive perspective. Ecol. Lett. 7, 734–739 10.1111/j.1461-0248.2004.00618.x (doi:10.1111/j.1461-0248.2004.00618.x)

[12] Réale D Martin J Coltman DW Poissant J Festa-Bianchet M 2009 Male personality, life-history strategies and reproductive success in a promiscuous mammal. J. Evol. Biol. 22, 1599–1607 10.1111/j.1420-9101.2009.01781.x (doi:10.1111/j.1420-9101.2009.01781.x) 19555442

[13] Nicolaus M Tinbergen JM Bouwman KM Michler SPM Ubels R Both C Kempenaers B Dingemanse NJ 2012 Experimental evidence for adaptive personalities in a wild passerine bird. Proc. R. Soc. B 279, 4885–4892 10.1098/rspb.2012.1936 (doi:10.1098/rspb.2012.1936) PMC3497238 23097506

[14] Dingemanse NJ Both C Drent PJ Tinbergen JM 2004 Fitness consequences of avian personalities in a fluctuating environment. Proc. R. Soc. Lond. B 271, 847–852 10.1098/rspb.2004.2680 (doi:10.1098/rspb.2004.2680) PMC1691663 15255104

[15] Adriaenssens B Johnsson JI 2013 Natural selection, plasticity and the emergence of a behavioural syndrome in the wild. Ecol. Lett. 16, 47–55 10.1111/ele.12011 (doi:10.1111/ele.12011) 23034098

[16] Smith BR Blumstein DT 2008 Fitness consequences of personality: a meta-analysis. Behav. Ecol. 19, 448–455 10.1093/beheco/arm144 (doi:10.1093/beheco/arm144)

[17] Both C Dingemanse NJ Drent PJ Tinbergen JM 2005 Pairs of extreme avian personalities have highest reproductive success. J. Anim. Ecol. 74, 667–674 10.1111/j.1365-2656.2005.00962.x (doi:10.1111/j.1365-2656.2005.00962.x)

[18] Quinn JL Patrick SC Bouwhuis S Wilkin TA Sheldon BC 2009 Heterogeneous selection on a heritable temperament trait in a variable environment. J. Anim. Ecol. 78, 1203–1215 10.1111/j.1365-2656.2009.01585.x (doi:10.1111/j.1365-2656.2009.01585.x) 19558612

[19] Schuett W Laaksonen J Laaksonen T 2012 Prospecting at conspecific nests and exploration in a novel environment are associated with reproductive success in the jackdaw. Behav. Ecol. Sociobiol. 66, 1341–1350 10.1007/s00265-012-1389-1 (doi:10.1007/s00265-012-1389-1)

[20] Duckworth RA Badyaev AV 2007 Coupling of dispersal and aggression facilitates the rapid range expansion of a passerine bird. Proc. Natl Acad. Sci. USA 104, 15 017–15 022 10.1073/pnas.0706174104 (doi:10.1073/pnas.0706174104) 17827278 PMC1986605

[21] Dingemanse NJ Réale D 2013 What is the evidence for natural selection maintaining animal personality variation? In Animal personalities: behaviour, physiology, and evolution (eds Carere C Maestripieri D), pp. 201–220 Chicago, IL: Chicago University Press

[22] Scales J Hymanb J Hughes M 2013 Fortune favours the aggressive: territory quality and behavioural syndromes in song sparrows, *Melospiza melodia*. Anim. Behav. 85, 441–451 10.1016/j.anbehav.2012.12.004 (doi:10.1016/j.anbehav.2012.12.004)

[23] van Overveld T Matthysen E 2010 Personality predicts spatial responses to food manipulations in free-ranging great tits (*Parus major*). Biol. Lett. 6, 187–190 10.1098/rsbl.2009.0764 (doi:10.1098/rsbl.2009.0764) 19906682 PMC2865041

[24] Verbeek MEM Drent PJ Wiepkema PR 1994 Consistent individual differences in early exploratory behavior of male great tits. Anim. Behav. 48, 1113–1121 10.1006/anbe.1994.1344 (doi:10.1006/anbe.1994.1344)

[25] Roulin A Dreiss AN Kölliker M 2010 Evolutionary perspective on the interplay between family life, and parent and offspring personality. Ethology 116, 787–796

[26] Duckworth RA 2006 Behavioral correlations across breeding contexts provide a mechanism for a cost of aggression. Behav. Ecol. 17, 1011–1019 10.1093/beheco/arl035 (doi:10.1093/beheco/arl035)

[27] McGlothlin JW Jawor JM Ketterson ED 2007 Natural variation in a testosterone-mediated trade-off between mating effort and parental effort. Am. Nat. 170, 864–875 10.1086/522838 (doi:10.1086/522838) 18171169

[28] Ketterson ED Nolan V Wolf L Ziegenfus C 1992 Testosterone and avian life histories—effects of experimentally elevated testosterone on behavior and correlates of fitness in the dark-eyed junco (*Junco hyemalis*). Am. Nat. 140, 980–999 10.1086/285451 (doi:10.1086/285451)

[29] Stoehr AM Hill GE 2000 Testosterone and the allocation of reproductive effort in male house finches (*Carpodacus mexicanus*). Behav. Ecol. Sociobiol. 48, 407–411 10.1007/s002650000247 (doi:10.1007/s002650000247)

[30] Wright J Cuthill I 1990 Biparental care: short-term manipulation of partner contribution and brood size in the starling, *Sturnus vulgaris*. Behav. Ecol. 1, 116–124 10.1093/beheco/1.2.116 (doi:10.1093/beheco/1.2.116)

[31] Houston AI Székely T McNamara JM 2005 Conflict between parents over care. Trends Ecol. Evol. 20, 33–38 10.1016/j.tree.2004.10.008 (doi:10.1016/j.tree.2004.10.008) 16701338

[32] Clutton-Brock TH 1991 The evolution of parental care. Princeton, NJ: Princeton University Press

[33] Dingemanse NJ De Goede P 2004 The relation between dominance and exploratory behavior is context-dependent in wild great tits. Behav. Ecol. 15, 1023–1030 10.1093/beheco/arh115 (doi:10.1093/beheco/arh115)

[34] Lambrechts MM 2004 Habitat quality as a predictor of spatial variation in blue tit reproductive performance: a multi-plot analysis in a heterogeneous landscape. Oecologia 141, 555–561 10.1007/s00442-004-1681-5 (doi:10.1007/s00442-004-1681-5) 15549399

[35] Perrins CM 1965 Population fluctuations and clutch size in the great tit, *Parus major* L. J. Anim. Ecol. 34, 601–647 10.2307/2453 (doi:10.2307/2453)

[36] Nur N 1984 The consequences of brood size for breeding blue tits. II. Nestling weight, offspring survival and optimal brood size. J. Anim. Ecol. 53, 497–517 10.2307/4530 (doi:10.2307/4530)

[37] Kempenaers B 1995 Polygyny in the blue tit: intra-sexual and inter-sexual conflicts. Anim. Behav. 49, 1047–1064 10.1006/anbe.1995.0134 (doi:10.1006/anbe.1995.0134)

[38] Patrick SC Browning LE 2011 Exploration behaviour is not associated with chick provisioning in great tits. PLoS ONE 6, e26383 10.1371/journal.pone.0026383 (doi:10.1371/journal.pone.0026383) 22028867 PMC3197650

[39] Drent PJ Marchetti C 1999 Individuality, exploration and foraging in hand raised juvenile great tits. In Proc. 22nd Int. Ornithology Congress, Durban (eds Adams NJ Slotow RH), pp. 896–914 Johannesburg, South Africa: BirdLife South Africa

[40] Herborn KA Macleod R Miles WTS Schofield ANB Alexander L Arnold KE 2010 Personality in captivity reflects personality in the wild. Anim. Behav. 79, 835–843 10.1016/j.anbehav.2009.12.026 (doi:10.1016/j.anbehav.2009.12.026)

[41] Schuett W Dall SRX Royle NJ 2011 Pairs of zebra finches with similar 'personalities’ make better parents. Anim. Behav. 81, 609–618 10.1016/j.anbehav.2010.12.006 (doi:10.1016/j.anbehav.2010.12.006)

[42] Schlicht L Girg A Loes P Valcu M Kempenaers B 2012 Male extrapair nestlings fledge first. Anim. Behav. 83, 1335–1343 10.1016/j.anbehav.2012.02.021 (doi:10.1016/j.anbehav.2012.02.021)

[43] Nicolaus M Bouwman KM Dingemanse NJ 2008 Effect of PIT tags on the survival and recruitment of great tits *Parus major*. Ardea 96, 286–292 10.5253/078.096.0215 (doi:10.5253/078.096.0215)

[44] Hurlbert SH 1984 Pseudoreplication and the design of ecological field experiments. Ecol. Monogr. 54, 187–211 10.2307/1942661 (doi:10.2307/1942661)

[45] Dingemanse NJ Both C Drent PJ Van Oers K Van Noordwijk AJ 2002 Repeatability and heritability of exploratory behaviour in great tits from the wild. Anim. Behav. 64, 929–938 10.1006/anbe.2002.2006 (doi:10.1006/anbe.2002.2006)

[46] Mutzel A Blom M Spagopoulou F Wright J Dingemanse NJ Kempenaers B 2013 Temporal trade-offs between nestling provisioning and defence against nest predators in blue tits. Anim. Behav. 85, 1471–1481 10.1016/j.anbehav.2013.03.043 (doi:10.1016/j.anbehav.2013.03.043)

[47] Schwagmeyer PL Mock DW 2003 How consistently are good parents good parents? Repeatability of parental care in the house sparrow, *Passer domesticus*. Ethology 109, 303–313 10.1046/j.1439-0310.2003.00868.x (doi:10.1046/j.1439-0310.2003.00868.x)

[48] Hadfield JD 2010 MCMC methods for multi-response generalized linear mixed models: the MCMCglmm R package. J. Stat. Softw. 33, 1–22 See http://www.jstatsoft.org/v33/i02/20808728

[49] R Development Core Team 2012 R: a language and environment for statistical computing. Vienna, Austria: R Foundation for Statistical Computing

[50] Nakagawa S Schielzeth H 2010 Repeatability for Gaussian and non-Gaussian data: a practical guide for biologists. Biol. Rev. 85, 935–956 20569253 10.1111/j.1469-185X.2010.00141.x

[51] Grace JB 2006 Structural equation modeling and natural systems. Cambridge, UK: Cambridge University Press

[52] Fox J Byrnes J, with contributions from Culberston M Friendly M Kramer A Monette G 2012 sem: Structural equation models. R package version 3.0-0 ed http://CRAN.R-project.org/package=sem

[53] Teplitsky C Mouawad NG Balbontin J de Lope F Moller AP 2011 Quantitative genetics of migration syndromes: a study of two barn swallow populations. J. Evol. Biol. 24, 2025–2039 10.1111/j.1420-9101.2011.02342.x (doi:10.1111/j.1420-9101.2011.02342.x) 21707815

[54] Dingemanse NJ Dochtermann N Wright J 2010 A method for exploring the structure of behavioural syndromes to allow formal comparison within and between data sets. Anim. Behav. 79, 439–450 10.1016/j.anbehav.2009.11.024 (doi:10.1016/j.anbehav.2009.11.024)

[55] Scheiner SM Donohue K Dorn LA Mazer SJ Wolfe LM 2002 Reducing environmental bias when measuring natural selection. Evolution 56, 2156–2167 12487346 10.1111/j.0014-3820.2002.tb00140.x

[56] Kontiainen P Pietiäinen H Huttunen K Karell P Kolunen H Brommer JE 2009 Aggressive ural owl mothers recruit more offspring. Behav. Ecol. 20, 789–796 10.1093/beheco/arp062 (doi:10.1093/beheco/arp062)

[57] Hollander FA Van Overveld T Tokka I Matthysen E 2008 Personality and nest defence in the great tit (*Parus major*). Ethology 114, 405–412 10.1111/j.1439-0310.2008.01488.x (doi:10.1111/j.1439-0310.2008.01488.x)

[58] Lima SL 2009 Predators and the breeding bird: behavioral and reproductive flexibility under the risk of predation. Biol. Rev. 84, 485–513 10.1111/j.1469-185X.2009.00085.x (doi:10.1111/j.1469-185X.2009.00085.x) 19659887

[59] Westneat DF Sargent RC 1996 Sex and parenting: the effects of sexual conflict and parentage on parental strategies. Trends Ecol. Evol. 11, A87–A91 10.1016/0169-5347(96)81049-4 (doi:10.1016/0169-5347(96)81049-4) 21237768

[60] McNamara JM Gasson CE Houston AI 1999 Incorporating rules for responding into evolutionary games. Nature 401, 368–371 10.1038/43869 (doi:10.1038/43869) 10517633

[61] Winkler DW 1987 A general model for parental care. Am. Nat. 130, 526–543 10.1086/284729 (doi:10.1086/284729)

[62] Sheldon BC 2000 Differential allocation: tests, mechanisms and implications. Trends Ecol. Evol. 15, 397–402 10.1016/S0169-5347(00)01953-4 (doi:10.1016/S0169-5347(00)01953-4) 10998516

[63] Møller AP Thornhill R 1998 Male parental care, differential parental investment by females and sexual selection. Anim. Behav. 55, 1507–1515 10.1006/anbe.1998.0731 (doi:10.1006/anbe.1998.0731) 9641996

[64] Wright J Both C Cotton PA Bryant D 1998 Quality versus quantity: energetic and nutritional trade-offs in parental provisioning strategies. J. Anim. Ecol. 67, 620–634 10.1046/j.1365-2656.1998.00221.x (doi:10.1046/j.1365-2656.1998.00221.x)

[65] Grieco F 2001 Short-term regulation of food-provisioning rate and effect on prey size in blue tits, *Parus caeruleus*. Anim. Behav. 62, 107–116 10.1006/anbe.2001.1736 (doi:10.1006/anbe.2001.1736)

[66] Naef-Daenzer L Naef-Daenzer B Nager RG 2000 Prey selection and foraging performance of breeding Great Tits *Parus major* in relation to food availability. J. Avian Biol. 31, 206–214 10.1034/j.1600-048X.2000.310212.x (doi:10.1034/j.1600-048X.2000.310212.x)

[67] Boon AK Réale D Boutin S 2007 The interaction between personality, offspring fitness and food abundance in North American red squirrels. Ecol. Lett. 10, 1094–1104 10.1111/j.1461-0248.2007.01106.x (doi:10.1111/j.1461-0248.2007.01106.x) 17877738

[68] Wolf M Van Doorn GS Leimar O Weissing FJ 2007 Life-history trade-offs favour the evolution of animal personalities. Nature 447, 581–584 10.1038/nature05835 (doi:10.1038/nature05835) 17538618

[69] Biro PA Stamps JA 2008 Are animal personality traits linked to life-history productivity? Trends Ecol. Evol. 23, 361–368 10.1016/j.tree.2008.04.003 (doi:10.1016/j.tree.2008.04.003) 18501468

[70] Schuett W Tregenza T Dall SRX 2010 Sexual selection and animal personality. Biol. Rev. 85, 217–246 10.1111/j.1469-185X.2009.00101.x (doi:10.1111/j.1469-185X.2009.00101.x) 19922534

[71] Gabriel PO Black JM 2012 Behavioural syndromes, partner compatibility and reproductive performance in Steller's jays. Ethology 118, 76–86 10.1111/j.1439-0310.2011.01990.x (doi:10.1111/j.1439-0310.2011.01990.x)

[72] Koolhaas JM 1999 Coping styles in animals: current status in behavior and stress-physiology. Neurosci. Biobehav. Rev. 23, 925–935 10.1016/S0149-7634(99)00026-3 (doi:10.1016/S0149-7634(99)00026-3) 10580307

[73] Dingemanse NJ Dochtermann N 2013 Quantifying individual variation in behaviour: mixed-effect modelling approaches. J. Anim. Ecol. 82, 39–54 10.1111/1365-2656.12013 (doi:10.1111/1365-2656.12013) 23171297

[74] Dochtermann NA Roff DA 2010 Applying a quantitative genetics framework to behavioural syndrome research. Phil. Trans. R. Soc. B 365, 4013–4020 10.1098/rstb.2010.0129 (doi:10.1098/rstb.2010.0129) 21078653 PMC2992739

